# Tumor microenvironment in osteosarcoma: From cellular mechanism to clinical therapy

**DOI:** 10.1016/j.gendis.2025.101569

**Published:** 2025-02-20

**Authors:** Yihan Yu, Kanglu Li, Yizhong Peng, Zhicai Zhang, Feifei Pu, Zengwu Shao, Wei Wu

**Affiliations:** a Department of Orthopedics, Union Hospital, Tongji Medical College, Huazhong University of Science and Technology, Wuhan, Hubei 430022, China; b Department of Orthopedics, Wuhan Hospital of Traditional Chinese and Western Medicine (Wuhan No.1 Hospital), Tongji Medical College, Huazhong University of Science and Technology, Wuhan, Hubei 430022, China

**Keywords:** Extracellular matrix, Immune inflammatory cells, Mesenchymal stem cell, Osteosarcoma, Tumor microenvironment

## Abstract

Osteosarcoma, a prevalent primary malignant bone tumor, predominantly affects both elderly and adolescent populations and usually has an unfavorable prognosis. The specific mechanisms underlying its invasive progression remain unclear. The tumor microenvironment includes not only cancer cells but also bone-related cells, immune cells, tumor-associated nerve cells, and cell-secreted factors. The cooperative and competitive interactions among these cellular components contribute to the proliferation, progression, metastasis, and immune evasion of osteosarcoma. Alterations in bone-related cells, resulting from oncogenic changes, can rapidly increase bone density or aggravate bone loss, thereby promoting the survival of osteosarcoma cells. During the progression of osteosarcoma, genetic alterations in tumor cells lead to changes in extracellular matrix components, influencing the variation in cell-secreted factors, promoting immunosuppression within the tumor microenvironment, and consequently affecting tumor proliferation and progression. This review summarizes the roles of tumor microenvironment components in the pathogenesis of osteosarcoma and discusses existing therapeutic targets. The findings suggest potential research directions for further investigation of osteosarcoma, provide novel insights into the development of osteosarcoma, and may guide the development of more effective anti-tumor strategies.

## Introduction

Cancer, a complex pathological entity, drives neoplastic transformation through genetic alterations at the cellular level. This process profoundly affects the surrounding tissues, giving rise to intricate vascular networks and establishing a hypoxic microenvironment. Concurrently, cancer spreads to adjacent and distant tissues and organs via vascular, lymphatic, or local diffusion pathways. The involvement of neural and immune organs indicates the systemic impact of cancer on the entire organism. Osteosarcoma (OS), a primary tumor originating in bone tissue, encompasses a spectrum ranging from benign to malignant, including intermediate subtypes. WHO classifies OS into subtypes based on location, histology, and genetics. These are divided into central (medullary) and surface (peripheral) tumors, which have multiple subtypes.^1^^,^^2^ Central (medullary) OS subtypes are conventional, telangiectatic, low-grade, and small-cell. Surface (peripheral) OS subtypes are periosteal, parosteal, and high-grade. Conventional central OS cells are known to produce osteoid, which contributes to a high degree of tissue necrosis. On imaging, the lesion often presents as an osteolytic process with cortical destruction, which subsequently invades the surrounding soft tissues, forming a Codman's triangle.^1^^,^^3^ Peripheral OS frequently develops at a slow rate, originating from the bone cortex and forming ossified foci or bone-like masses on the bone surface or in close proximity to the bone.^4^^,^^5^ It is less aggressive than central OS and typically has a more favorable prognosis.^6^^,^^7^ Malignant OS commonly occurs in children and elderly individuals. The five-year survival rate of patients with OS is approximately 60%, with recurrence and metastasis rates of 20%. In contrast to other bone tumors, OS is predominantly treated with surgical intervention supplemented by chemotherapy and neoadjuvant therapy in clinical settings. However, the prognosis of OS has not significantly improved over the past three decades, and overcoming chemotherapy resistance is challenging.^8^^,^^9^ Moreover, many patients develop extensive tumor metastasis and recurrence.^10^^,^^11^ Irrespective of its origin, OS, characterized by heterogeneous cellular interactions, undergoes proliferation and progression largely based on the interplay between genetically altered tumor cells and their surrounding microenvironment, similar to other neoplastic cell populations.

During the progression of cancer, the tumor microenvironment (TME) emerges as a dynamic milieu resulting from close interactions between tumor cells and the host. TME comprises not only cells, tumor microvessels, and nerves but also microbial communities, cellular factors, exosomes, and metabolic products secreted by microenvironmental cells.^12^ It is generated, continuously modified, and predominantly orchestrated by tumors, coordinating molecular and cellular events occurring in the surrounding tissues.^13^ Previous studies have reported that OS cells in isolation inadequately explain their primary growth and metastasis. The clinical manifestations of OS collectively rely on the reciprocal interactions between differentiated tumor cells and neighboring stromal cells. As an important site of tumor metabolism, TME is regulated by both intrinsic factors within tumor cells and external metabolic products in the microenvironment. This regulation involves intra-tumoral cell metabolism, interactions between tumor cells and surrounding non-tumor cells locally and distantly, and maintenance of host homeostasis by tumor cells. Cancer cells use various metabolic pathways to fulfill their increasing bioenergetic and biosynthetic demands and oxidative stress required for proliferation and survival.^14^ Abnormal accumulation of cellular factors or metabolic products in the TME may promote tumorigenesis. Owing to nutrient depletion in the TME, cancer cells stimulate mechanisms for nutrient production, thereby sustaining tumor proliferation.^15^ These biological behaviors contribute to morphological and molecular similarities between stromal and tumor cells in TME, posing challenges in distinguishing them during clinical biopsy and hindering independent studies. Given the difficulty in improving prognosis solely by targeting OS cells, numerous scholars have redirected their focus toward investigating the TME of OS. Recent studies have suggested the presence of interactions between nerves and immune cells within the TME.^16^ Consequently, existing studies tend to focus on the role of osteoblasts and immune cells in the OS microenvironment, to elucidate the mechanisms underlying tumor metastasis and drug resistance.^17^, ^18^, ^19^ However, this approach has not fully addressed the intricate phase-action relationship between OS cells and the microenvironment.

The specific and functional structure of bone tissue has led to an increased focus on the OS microenvironment among researchers. This review provides a comprehensive overview of the OS microenvironment, delineating the cellular and non-cellular constituents. It presents a synthesis of the existing research on bone-associated cells and immune cells in the OS microenvironment and explores emerging areas of investigation, including extracellular vesicles (EVs) and neuronal cells. The findings will inform the identification of promising research avenues and the development of therapeutic strategies for OS in the clinical setting.

## Facts

1. The OS microenvironment exhibits a diverse array of components that significantly influence tumor invasion and progression.
2. Among the constituents of the OS microenvironment, bone-related cells play a crucial role in the malignant transformation of OS.
3. The precise mechanisms underlying the impact of neuro-tumor cross-talk within the TME on the malignancy of OS remain incompletely elucidated.
4. Exosomes serve as a mode of cross-talk between OS cells and the TME, influencing tumor invasion.

## Bone-associated cells

Osteoblasts, osteocytes, and osteoclasts constitute vital components of the TME of OS. In a healthy organism, the equilibrium among these bone-related cells regulates the emergence of bone tumors. However, when these cells undergo oncogenic alterations, the abnormal activation of specific cells can rapidly induce highly concentrated bone density or bone loss within a short period, creating a microenvironment conducive to tumor cell survival. Consequently, modulating the balance among bone-related cells represents a promising strategy for suppressing tumor progression.

## Osteoblasts

OS predominantly develops in the central regions of bones, resulting in abnormal bone formation. Based on cellular subtypes, OS can be classified as osteoblastic, chondroblastic, and fibroblastic. Osteoblasts play an important role in the initiation and metastasis of OS.^20^^,^^21^ Osteoblastic OS represents a major subtype in the context of both primary and metastatic tumors. Osteoblasts enhance the activity of differentiation markers within the TME by secreting various cytokines, such as interleukin (IL)-6. RUNX family transcription factor 2 (RUNX2), an essential transcription factor for consistent osteoblast differentiation, regulates the cell cycle of osteoblasts, thereby contributing to the pathogenesis of OS.^22^ Osteoblasts not only play a crucial role in bone matrix production but also secrete proteinases and matrix metalloproteinases (MMPs). In addition, they interact with osteoclasts in most OS subtypes. Before osteoclasts absorb the mineralized bone matrix, endosteal osteoblasts reshape the non-mineralized bone crown, leading to surface retraction. This process necessitates the activity of proteolytic enzymes derived from osteoblasts.^23^^,^^24^ MMPs located on the surface of osteoblasts can influence the malignant resorption cycle. Although MMPs within the host do not impair osteoclast activity, MMP-2 derived from osteoblasts contributes to tumor cell survival within the TME by activating latent transforming growth factor beta (TGFβ).

RUNX2, a transcription factor involved in the differentiation and migration of osteoblasts and chondrocytes, activates transcriptional activity through the phosphoinositide 3-kinase (PI3K)/protein kinase B (Akt) pathway, thereby promoting tumor progression and invasion.^25^ Villanueva et al found that surface-expressed RUNX2 on osteoblasts can regulate the expression and secretion of the matricellular protein osteonectin in OS cells. This regulatory effect enhances the adhesion of OS cells to pulmonary endothelial cells, thereby influencing the pulmonary metastasis of OS.^26^ On comparing murine and human models of OS, Lu et al found that up-regulated RUNX2 expression was predominantly associated with the osteoblastic subtype in human models but the chondroblastic subtype in mouse models.^27^ Concurrently, they established various mouse models with or without the knockout of RUNX2, elucidating different expression patterns of marker genes. These findings improve the understanding of the pathogenesis of RUNX2-associated OS. Furthermore, Chan et al suggested the potential involvement of the Hedgehog signaling pathway in the role of mature osteoblasts in OS.^28^ They enhanced the malignancy of OS by up-regulating Hedgehog signaling in mature osteoblasts in a mouse model with a heterozygous background of P53. Inhibiting the Hedgehog signaling pathway not only suppressed the maturation of osteoblasts but also inhibited tumor progression.

## Osteoclasts

Cancer-induced bone degradation is an integral part of the pathological processes of both primary and metastatic OS. This process involves the osteoclast-mediated destruction of inorganic bone components and the dissolution of collagen and other related factors.^29^ Osteoclasts, a type of bone cell, play an essential role in promoting bone resorption and absorption. They originate from terminally differentiated multinuclear cells within the mononuclear macrophage system. The production of osteoclasts is intricately regulated by various factors, including transcription factors, cytokines, and lymphocytes. These regulatory molecules collectively facilitate the activation of transcription factors and the expression of downstream genes in osteoclasts. Receptor activator of nuclear factor kappa-B ligand (RANKL), a key factor of osteoclasts, exhibits high expression in lytic bone tumors and promotes the continuous proliferation and differentiation of osteoclasts. However, in osteoblastic bone tumors, RANKL is expressed in osteoblasts and inhibits surface membrane receptors on osteoclasts.^30^ Osteoclasts release various growth factors, such as TGFβ, at bone resorption sites, stimulating tumor cell proliferation and eventually establishing a vicious cycle between tumor growth and bone absorption.^31^, ^32^, ^33^ Chondroblastic, recurrent, and lung metastatic OS lesions have lower osteoclast infiltration than primary osteoblastic OS lesions.^34^

Osteoclast genesis relies on the RANK–RANKL signaling pathway. Loss of RANK expression in marrow cells leads to a deficiency in osteoclast precursors and mature osteoclasts.^35^ Chen et al and Molyneux et al demonstrated that eliminating osteoclasts delayed the onset of tumors and reduced the likelihood of lung metastasis in mice.^35^^,^^36^ In a recent study, osteoclasts from patients with OS were found to have elevated activity of tartrate-resistant acid phosphatase (TRAP). EVs derived from OS cells contained miR-501-3p, which promoted osteoclast differentiation and exacerbated bone loss through two pathways.^37^ First, the vesicles promoted osteoclast differentiation by transferring miR-501-3p to bone marrow-derived mononuclear cells, thereby leading to bone loss indirectly. Second, they directly promoted osteoclast differentiation by acting on the PTEN (phosphatase and tensin homolog)/PI3K/Akt pathway. Gomez et al experimentally validated that zoledronic acid exerted therapeutic effects against OS by targeting osteoclasts and preserving macrophage function.^38^ In addition, they found that osteoclasts and OS cells might collectively participate in bone degradation. Lars et al found that sarcoma cells degraded crucial membrane-associated proteins involved in bone remodeling through MMPs. Inhibition of MMPs and the binding of two membrane proteins significantly suppressed the activity of sarcoma cells in bone remodeling.^39^^,^^40^ The effects of OS cells on osteoclasts are diverse and contradictory. Studies have shown that co-culturing human OS cells with human peripheral blood mononuclear cells leads to an increase in the number and resorption activity of osteoclasts.^41^ However, in a few studies, the number and mRNA levels of osteoclasts in immunohistochemical evaluations in OS patient biopsies were decreased.^42^ In clinical settings, the tumor-induced decrease in osteoclast number may be more pronounced and prognosis after chemotherapy may be less favorable in metastatic OS than in non-metastatic OS.^42^^,^^43^

The mechanisms underlying bone degeneration pathology and osteoclasts in OS have not been extensively investigated, as in the case of bone metastasis. This lack of focus may be attributed to the lower incidence of osteolytic OS, the differential distribution of cells in patients with OS, and the fact that OS accounts for <1% of all cancer diagnoses.^44^^,^^45^ However, characterizing the expression patterns of osteoclast and osteoblast markers may improve the understanding of tumor progression in OS.

The roles of bone-related cells of OS TME are summarized in Table 1.

**Table 1:** Bone-related cells in the tumor microenvironment of osteosarcoma.

Component	Origin	Functions in the tumor microenvironment of osteosarcoma
Osteoblasts	Bone	Regulate tumor metastasis and spread^20^^,^^21^ Promote the formation and maintenance of microenvironment matrix^26^ Participate in bone changes together with osteoclasts^23^^,^^24^
Osteoclasts	Terminally differentiated multinuclear cells within the mononuclear macrophage system	Affect the proliferation and invasion of tumor cells^31^ Regulate tumor metastasis and spread^31^, ^32^, ^33^, ^34^, ^35^, ^36^ Participate in bone changes together with osteoblasts^38^ Affect the prognosis of tumors after chemotherapy^42^^,^^43^

## Extracellular matrix components in OS

### Extracellular matrix

The extracellular matrix (ECM) is primarily composed of collagen and plays a crucial role in providing structural support to cells, promoting cell adhesion, storing and transporting cell nutrients, and transmitting cell signals.^46^ Dysregulation of ECM is a significant characteristic of tumor progression in OS. Genetic alterations in tumor cells may alter the composition and properties of ECM, leading to carcinogenic transformation.^47^

### Structural proteins

Structural proteins within the ECM predominantly include collagen and elastin. Collagen, the most abundant protein in TME, dynamically reshapes the structure of TME and influences tumor progression. As a crucial source of nutrients, collagen plays an important role in regulating growth and providing immune signals for tumor cell proliferation. MMPs cleave collagen, generating specific collagen fragments. These fragments can be detected in the blood of patients with cancer and are associated with an unfavorable prognosis. Leukocyte-associated immunoglobulin-like receptor-1 (LAIR-1), an immunosuppressive collagen receptor, can be detected in both TME and blood and triggers the inhibition of CD3 signal transduction and interferon-gamma (IFN-γ) secretion in T cells.^48^ Collagen deposition in TME promotes tumor progression and metastasis in most cancer types, including OS. Zhang et al demonstrated that overexpression of collagen type VI alpha 1 chain (COL6A1) in TME inhibited the activation and expression of signal transducer and activator of transcription 1 (STAT1) through ubiquitination and proteasomal degradation, thereby promoting the migratory and invasive abilities of OS cells.^49^ Additionally, COL6A1 can drive the transformation of normal fibroblasts to cancer-associated fibroblasts (CAFs) by secreting pro-inflammatory cytokines. Activated CAFs mediate the TGFβ/COL6A1 signaling pathway, promoting OS cell invasion and migration. Wang et al found that the expression of collagen β(1-O) galactosyltransferase 2 (COLGALT2) was significantly higher in metastatic OS than in primary OS.^50^ In addition, researchers have designed collagen-based biochemical scaffolds to inhibit the growth of OS cells and promote the recovery of surrounding tissues.^51^, ^52^, ^53^

Elastic proteins, another dynamic component of ECM, have been reported to change tumor progression and metastasis in breast cancer and oral squamous cell carcinoma.^54^, ^55^, ^56^ The lysyl oxidase (LOX) family is responsible for the covalent cross-linking of collagen and elastin, maintaining the stability of ECM. Abnormal expression or activity of LOX family members may alter the cross-linking of structural proteins, promote TME remodeling, and facilitate tumor invasion, metastasis, proliferation, and apoptosis in several cancer types.^57^ Uma et al performed proteomic analysis on samples from elderly patients with OS and found that increased expression of HSP90, elastin microfibril interface-located protein 1 (EMILIN1), and clusterin was closely associated with tumor invasion and drug resistance.^58^ However, studies assessing the role of structural proteins in OS are limited. Investigating the role of structural proteins in the pulmonary metastasis of OS, particularly in cases with a high rate of metastasis, may contribute to further clinical research.

### Fibronectin

Fibronectin is a multifunctional ECM protein and a high-molecular-weight glycoprotein. It binds to various components on the cell surface and within ECM, including collagen, fibronectin, heparin, and myosin, and participates in cell adhesion, migration, tumor progression, and other related processes in dimeric or polymeric forms.^59^ Fibronectin serves as an indicator of malignant transformation in OS.^60^

Pavlou et al constructed a novel three-dimensional OS model. They introduced fibronectin, marrow adhesive protein, and bone granules to the tri-culture system to investigate the impact of the biomimetic matrix on tumor cell behavior. The results showed increased migration of OS cell aggregates from the bone-like matrix to the surrounding acellular marrow-like ECM.^61^ These findings suggest that fibronectin influences the invasive ability of OS cells within TME. Dedhar et al found that the expression of a fibronectin receptor was up-regulated in drug-resistant MG-63 OS cells. This finding indicates the ability of OS cells to alter their phenotype regarding fibronectin receptor quantity under conditions of drug resistance.^62^ Numerous cytokines regulate fibronectin in the TME of OS. Wang et al reported that vitronectin up-regulated the expression of fibronectin and MMP2, significantly promoting the migratory and invasive abilities of OS MG-63 and HOS cells *in vitro*. Ruoslahti et al reported that the fibronectin-binding integrin α5β1 present in the TME played an important role in promoting tumor cell invasion in OS, melanoma, and glioblastoma.^63^

Pulmonary metastasis is a common outcome of advanced OS. The adhesion of OS cells to ECM via integrins plays a crucial role in pulmonary metastasis. Fibronectin and its derivatives can be targeted to attenuate tumor migration. Pasqualini et al demonstrated that fibronectin polymerization significantly alleviated experimental and spontaneous lung metastasis in both *in vitro* and *in vivo* models of human OS.^64^ Therefore, targeting fibronectin may represent a promising strategy for preventing tumor migration and metastasis.

### Hyaluronic acid

Hyaluronic acid, a natural glycosaminoglycan, possesses excellent water solubility, biocompatibility, and degradability. Hyaluronic acid and its chemically modified derivatives have been used as drug carriers in the treatment of various diseases. They enable the controlled release of different types of drugs, including proteins, nucleic acids, and anti-tumor agents. Given that TME exhibits specific physiological regulation in terms of pH, redox potential, temperature, and enzymes, it serves as a promising trigger for drug release.^65^, ^66^, ^67^, ^68^ Owing to their specific binding affinity for various receptors on tumor cell surfaces, hyaluronic acid and its derivatives can be used for targeted drug delivery in anti-tumor therapy.^69^

In recent years, many researchers have designed targeted drug delivery systems incorporating hyaluronic acid for the treatment of OS. Xu et al engineered zoledronic acid-loaded nanoparticles comprising hyaluronic acid, polyethylene glycol, and hydroxyapatite. These nanoparticles increased the expression of apoptosis-related proteins and blocked the cell cycle of OS cells, preventing the local recurrence of OS.^70^^,^^71^ Yu et al developed a hydrogel loaded with curcumin/methacrylated hyaluronic acid (HAMA)/silk fibroin for OS treatment and bone regeneration. They prolonged drug release by stabilizing the cumulative release curve, thereby enhancing the long-term therapeutic efficacy and preventing the recurrence of OS.^72^ Chianese et al used hyaluronic acid to recognize CD44, which is highly expressed in OS cells. They modified ZrO2–acetylacetone nanoparticles with hyaluronic acid to induce apoptosis in OS cells through reactive oxygen species.^73^ Altogether, these studies indicate that hyaluronic acid is a crucial tool for constructing sustained-release drug delivery systems. Specific targeted drugs can be encapsulated in these systems to repair bone defects in OS. As a novel carrier, hyaluronic acid demonstrates significant potential for targeted drug delivery in OS.

### Cancer-associated fibroblasts

The ECM of TME comprises various components, including proteins and cellular components such as CAFs. Among TME-resident stromal cells, CAFs represent one of the most abundant cell types. They influence tumor cells and stroma through direct contact or paracrine secretion of cytokines, thereby facilitating ECM remodeling and affecting cancer cell behavior, epithelial–mesenchymal transition, and drug resistance.^74^, ^75^, ^76^ During tumor progression, CAFs can generate and accumulate a substantial amount of ECM in the TME, forming a capsule-like structure that envelops cancer cells. CAFs actively use the contractile force of actin to compress cancer cells, modulate mechanical signal transduction, and reshape the morphological features of tumors.^77^

Fibroblast activation protein and α-smooth muscle actin serve as markers of activated fibroblasts. These positive markers suggest an association between CAFs and various pro-tumorigenic functions, including tumor initiation, angiogenesis, immune suppression, and metastasis.^78^ Mesenchymal stem cells (MSCs) can undergo epithelial–mesenchymal transition and transform into CAFs. Knockout of G protein-coupled receptor 68 (GPR68) or inhibition of the IL-6/STAT3 pathway in MSCs has been shown to suppress *in situ* OS growth and extend the lifespan after tumor xenograft transplantation. Lin et al demonstrated that treating bone marrow-derived MSCs with a conditioned medium from OS cell lines increased IL-6 expression and STAT3 phosphorylation and triggered the transformation of MSCs to CAFs, thereby promoting cell proliferation, migration, and invasion.^79^^,^^80^ Therefore, targeting CAF-specific positive markers in TME serves as a promising therapeutic strategy for OS. Simultaneously, there is an ongoing exploration of addressing the changes in the TME resulting from cancer-stroma cell reprogramming through combined treatment of immune TME and CAFs.

TME-resident CAFs play an important role in tumor proliferation and progression. Specific pathways and mechanisms involved in the progression of OS have not yet been identified, which may represent a potential avenue for further research on anti-cancer therapy. In-depth research into the intricate interactions between CAFs and the immune microenvironment, especially the complex crosstalk between CAFs and immune cells, may guide the development of new strategies for targeted immunotherapy.^81^

### Mesenchymal stem cells

MSCs are non-hematopoietic progenitor stem cells found within the bone marrow cavity. Similar to other stem cells, MSCs play a crucial role in maintaining the regeneration of various tissues while ensuring pluripotent induction and differentiation. Specific stimulating factors can be used to stimulate the differentiation of MSCs into osteoblasts, chondrocytes, and adipocytes *in vitro*.^82^ MSCs exert immunomodulatory effects through cell-to-cell contact or secretion of factors that interact with cells of the innate and adaptive immune systems.^83^ With regard to surface markers, MSCs exhibit positive expression of CD73/CD90/CD105 and lack the expression of CD45/CD34/CD14.^84^ The expression of these surface markers is altered upon exposure to factors secreted by invasive OS cells. Brune et al showed that when CD271-positive OS cells acquired all characteristics of aggressive cancer stem cells, MSCs from a consistent source were negative for CD127.^19^

The role of MSCs in either promoting or inhibiting tumor progression remains controversial.^85^ Ohlsson et al showed that MSCs suppressed the proliferation and migration of colorectal cancer cells when co-cultured with an equivalent number of cancer cells or when their number exceeded that of cancer cells.^86^ However, Perrot et al observed instances of local recurrence of OS 18 months after autologous fat transplantation, suggesting that the high concentration of MSCs in adipose tissue was the potential cause of recurrence.^87^ OS cells can enhance the proliferation of MSCs and reduce their differentiation potential, thereby enhancing tumor growth.^88^ MSCs can promote the proliferation and metastasis of OS *in vivo*.^89^ Numerous studies have demonstrated that the migratory ability of MSCs is associated with the occurrence, progression, and pulmonary metastasis of OS.^90^, ^91^, ^92^

Cytokines and chemokine receptors, mainly C-X-C motif chemokine receptor 4 (CXCR4), contribute to the enhanced migratory and invasive abilities of MSC-dependent OS. Inhibition of CXCR4 disrupts the crosstalk between OS cells and ECM, significantly suppressing tumor cell migration.^93^ *In vitro* studies have demonstrated that CXCR4 regulates the production of vascular endothelial growth factor (VEGF) by MSCs and surface MSCs can promote the growth of OS through VEGF-induced CXCR4 signaling.^92^ The positive expression of CXCR4/VEGF serves as an indicator of tumor metastasis and progression and is associated with a low survival rate.^94^ Additionally, nutritional factors secreted by MSCs, such as C–C motif chemokine ligand 5 (CCL5) and aquaporin 1 (AQP1), can promote tumor migration and invasion.^95^^,^^96^ IL-6, a cytokine overexpressed in many tumors, exhibits pro-tumorigenic activity in OS. It can enhance chemotherapy resistance, induce osteoclast activity at the tumor site, and enhance tumor cell invasion in OS.^45^ Notably, IL-6 is not secreted by OS cells but by cancer-activated stromal cells. It directly promotes tumor growth by activating the Janus kinase (JAK)/STAT3 pathway and influences prognosis.^97^, ^98^, ^99^ Recent studies have indicated that MSCs can affect OS cells by secreting exosomes.^97^^,^^100^ Exosomes are small lipid-bilayer-membrane-bound EVs that encapsulate various bioactive substances, including microRNAs (miRNAs), proteins, and cytokines, and mediate their transfer between MSCs and OS cells. In a study, exosomes were used to deliver miR-101 for the treatment of metastatic OS.^101^ In particular, administration of exosomes containing miR-101 decreased the number of metastatic pulmonary nodules in mice with metastatic OS, without causing evident side effects. Baglio et al demonstrated that OS-derived exosomes selectively incorporated membrane-associated TGFβ and stimulated MSCs to release IL-6. Blocking TGFβ signaling with a TGFβ type I inhibitor significantly reduced the release of IL-6 from MSCs.^97^

However, these findings do not validate that MSCs promote the growth of OS. Normal, non-malignant MSCs can be isolated from the microenvironment of OS using culture techniques. An in-depth understanding of the mechanisms underlying the activity of MSCs may help to regulate CXCR4/VEGF and IL-6 selectively. In addition, inhibiting the cytokine-induced homing of stromal cells to the tumor site represents a promising strategy for the clinical treatment of OS. Altogether, understanding the role of MSCs within OS TME may guide the development of novel, more effective targeted therapies.

The ECM and related cells of OS TME are summarized in Table 2.

**Table 2:** Extracellular matrix and related cells in the tumor microenvironment of osteosarcoma.

Component		Origin	Functions in the tumor microenvironment of osteosarcoma
Extracellular matrix	Structural proteins	Tumor microenvironment	Reshape the structure of tumor microenvironment and maintain the morphological characteristics of the microenvironment^51^, ^52^, ^53^ Affect the proliferation and invasion of tumor cells^49^ Regulate tumor metastasis and spread Affect tumor immune signaling^58^
	Fibronectin	Tumor microenvironment	Maintain the morphological characteristics of the tumor microenvironment Affect tumor proliferation and invasion^59^ Regulate tumor metastasis and spread^61^^,^^63^^,^^64^
	Hyaluronic acid	Tumor microenvironment	Regulate the physical and chemical properties of the tumor microenvironment^65^, ^66^, ^67^, ^68^ Maintain intercellular material transfer^69^, ^70^, ^71^, ^72^, ^73^
Cancer-associated fibroblasts		Tumor microenvironment	Secrete extracellular matrix to maintain the external morphological characteristics of tumors^74^, ^75^, ^76^, ^77^ Affect the proliferation and invasion of tumor cells^78^ Regulate tumor metastasis and spread^79^^,^^80^ Inhibit body immune function^81^
Mesenchymal stem cells		Bone marrow cavity	Maintain the regeneration of various tissues and have the ability to induce differentiation of pluripotency^82^ Play the role of the body's immune function^83^^,^^84^ Affect the proliferation and invasion of tumor cells^88^ Regulate tumor metastasis and spread^89^, ^90^, ^91^, ^92^

### Immune inflammatory cells

As one of the primary sources of immune cells in the body, the bone marrow contains various immune cells, including T lymphocytes and macrophages, which are produced and transported to other immune organs. Under normal physiological conditions, immune cells actively monitor tumor cells and exert cytotoxic effects through immune reactions. However, in the distinctive pathological environment of OS, infiltrating tumor cells induce immune suppression, impeding the normal functioning of the immune system. In a study, whole-genome sequencing, RNA profiling, T-cell receptor sequencing, immunohistochemical analysis, and reverse-phase protein array analysis of 48 OS specimens revealed that immune cell infiltration levels were significantly lower in OS than in other types of tumors. In addition, T-cell receptor clonality and expression of new antigens were relatively low in OS.^102^ Therefore, reconstruction of immune cell infiltration is an important avenue for in-depth investigation of the onset and progression of cancer.

### T lymphocytes and B lymphocytes

Lymphocytes play a crucial role in maintaining the homeostasis of the human immune system and are essential for immune tolerance. In addition, they are involved in the regulation of anti-tumor immunity, serving as an important focus of cancer research. T lymphocytes are one of the predominant immune cell infiltrates in the TME of OS, second only to macrophages.^103^ Tumor-infiltrating lymphocytes can be detected in >70 % of patients with OS. OS cells can modulate the recruitment and differentiation of tumor-infiltrating immune cells, thereby establishing a local immune-tolerant environment conducive to their growth, progression, drug resistance, and metastasis.

Induced regulatory T cells are a subset of T cells, originating from peripheral naive T cells induced by signals from TME, including tumor antigens, cytokines, and other soluble molecules.^104^ T-cell receptor isolated from regulatory T cells exhibits specific immunogenicity against primary tumors and newly mutated antibodies. This phenomenon suggests that regulatory T cells within tumors can selectively influence the TME by promoting their activation and proliferation, and the derived T-cell receptor circulates within the tumor.^105^ Induced regulatory T cells use various mechanisms to facilitate tumor progression and inhibit the anti-tumor immune functions of T cells, natural killer cells, and dendric cells. These mechanisms include the secretion of inhibitory cytokines, the release of granule enzymes and perforins, and interference with cellular metabolism by affecting cytokines such as IL-2 in TME.^106^, ^107^, ^108^ Wu et al found that the immunogenomic landscape of OS exhibited complexity and significant disease heterogeneity. This genomic complexity does not contribute to a robust immune response but promotes an immune-inhibitory phenotype through multiple mechanisms.^102^ Therefore, these immune-inhibitory phenotypes may serve as potential therapeutic targets. Researchers have attempted to enhance the infiltration levels of T lymphocytes owing to their crucial role in the pathogenesis of OS. Caterina et al reported that administration of Toll-like receptor 9 (TLR9) agonists directly to mouse lesions significantly impaired local tumor growth, with the therapeutic effect extending to the untreated lesions on the opposite side. Flow cytometric analysis of the TME of OS after TLR9 treatment revealed a marked increase in the infiltration levels of activated CD8^+^ T cells in both lesions. CD8^+^ T cells did not suppress local tumor growth but induced T-cell receptor-specific clones in untreated lesions.^109^ These findings indicate that TLR9 agonists can induce systemic adaptive immune responses while inhibiting the growth of OS. Yang et al found that chimeric antigen receptor T-cell (CAR-T) therapy exhibited significant anti-tumor efficacy not only in hematologic malignancies but also in heterogeneous and metastatic OS without causing evident toxicity. They used tumor-targeted interleukin-12 (attIL12)-modified expanded T cells, which were anchored to the cell membrane, to inhibit cancer cells. However, this treatment method incurred a high cost for T cells. Therefore, therapeutic strategies should be aimed at hindering the growth of OS cells by inducing their terminal differentiation to bone-like cells instead of directly killing tumor cells using attIL12-modified peripheral blood mononuclear cells.^110^ NKG2D ligand (NKG2DL) is an antibody expressed on the surface of immunosuppressive cells within the TME. Fernández et al established CD45RA^−^ memory T cells expressing NKG2D-4-1BB-CD3z CAR and used them to target OS cells expressing NKG2DL, achieving high anti-tumor activity in NKG2D^−^CAR ^+^ memory T cells.^111^ Huang et al showed that injection of CAR-T cells targeting IL-1Rα successfully regressed the lung metastasis of OS in mice, with low cytotoxicity. The low percentage of CD8^+^ T cells in OS lung metastases observed in the study by He et al^112^ was associated with a diminished tumor cell-killing function. Additionally, due to the low expression of immune checkpoint genes, this group of patients is not a suitable candidate for immune checkpoint inhibitor therapy.

B cells, another crucial component of TME, significantly influence tumor initiation and progression. They secrete IFN-γ to sustain lymphatic function and exert anti-tumor effects by producing tumor-specific antibodies and enhancing the anti-tumor effects of natural killer cells and T cells. However, studies investigating the role of B cells in OS are limited, and whether B cells possess only tumor-suppressing activity remains unclear. B cells can promote cancer development by secreting IL-10 and inducing tumor angiogenesis and immunosuppression.^113^ To date, most studies have focused on B-cell lymphoma 2 (Bcl-2) and its related proteins. Inducing the expression of Bcl-2 through miRNA-targeted regulation of different pathways has been associated with tumor progression.^114^ However, some scholars have conducted genomic studies on B lymphocytes found in the TME of OS. Zhang et al identified 261 B-cell marker genes in OS through single-cell sequencing and constructed a prognostic model based on four marker genes (RPL37A, MEF2C, PLD3, and SNX2). In addition, they observed significant differences in immune infiltration between low- and high-risk groups and validated that the model had good predictive performance.^115^

Although expanded tumor-infiltrating lymphocytes exert stronger toxic effects on allogeneic and autologous tumor cells than on autologous peripheral lymphocytes, the construction of modified T lymphocytes is considered a feasible and effective approach to restoring the immunosuppressive phenotype of OS in most cases. Overexpressed programmed cell death ligand 1 (PD-L1) on OS cells can induce immunosuppression by binding to programmed death 1 (PD-1) on T cells, suggesting that PD-L1 is a promising target for blockade. Disrupting the binding between PD-L1 and PD-1 can reduce tumor burden, leading to prolonged survival. T cells can recruit other cytotoxic cells to suppress the proliferation, differentiation, and pulmonary metastasis of OS. Therefore, CAR-T therapy targeting antigens expressed on cancer cells is an effective therapeutic strategy for OS.

### Macrophages

Tumor-associated macrophages (TAMs) infiltrate solid tumors and play a crucial role in the occurrence and development of tumors.^116^^,^^117^ They have been shown to influence tumor growth, invasion, metastasis, angiogenesis, and immunosuppression. Under the regulation of different cytokines, TAMs undergo a phenotypic transition between cytotoxic M1-type macrophages that kill tumor cells and pro-tumor M2-type TAMs. The proportion of M2-type TAMs in TME is closely related to patient prognosis.^118^^,^^119^ Modulating the transition of TAMs from the M2 to the M1 phenotype is considered a potential therapeutic strategy for tumors.^120^ In OS, macrophages occupy a central node in the core of bone homeostasis and destruction, playing a fundamental role in the tumor immune environment of OS.

M1-type TAMs play a role in enhancing inflammatory signaling in primary OS. They recruit and modulate immune cells during induced malignant pleural effusion or tumor-associated inflammatory reactions by secreting IL-1B, C–C motif chemokine ligand 2 (CCL2), and C–C motif chemokine ligand 20 (CCL20). On the contrary, M2-type TAMs suppress T cell-mediated anti-tumor immunity through multiple signaling pathways, including the CD86/cytotoxic T-lymphocyte associated protein 4 (CTLA4) and secreted phosphoprotein 1 (SPP1)/CD44 pathways. Han et al showed that depletion of M2-type TAMs enhanced the proliferation of immunosuppressive T lymphocytes and the expression of pro-inflammatory cytokines in OS.^121^ M2-type TAMs exacerbate the immunosuppressive phenotype of T lymphocytes. Additionally, clinical studies have shown a correlation between M2-type TAMs and lung metastasis or other manifestations of OS. Zhang et al found that the immunosuppressive activity of M2-type TAMs contributed to malignant pleural effusion in OS.^122^ M2-type TAMs promote tumor proliferation, angiogenesis, and metastasis in OS.^123^ Zhou et al reported that all-trans-retinoic acid (ATRA) inhibited M2-type macrophages induced by IL-13 or IL-4.^124^ *In vitro* experiments showed that ATRA counteracted the positive effects of M2-type macrophages on the migration of OS cells and decreased the number of lung metastatic lymph nodes. In an *in situ* model, the number of M2-type macrophages was significantly low in metastatic lymph nodes. In addition, inhibition of the polarization of M2-type TAMs by ATRA resulted in the suppression of OS metastasis. The high abundance of M2-type TAMs in the TME of OS often results in a poor prognosis. Before chemotherapy, the number of CD68 TAMs in primary lesions is similar between patients with metastatic and non-metastatic OS. However, an imbalance in the M1/M2 ratio may result in increased infiltration of M2-type TAMs in TME, forming an immune-tolerant environment in both primary and metastatic lesions and affecting patient survival.^125^ Using single-cell sequencing, He et al show that macrophages in OS lung metastases lack distinct M1 or M2 polarization. This suggests that in the OS microenvironment, macrophages may exist in an unpolarized state, exhibiting intermediate polarization states between the M1 and M2 states.^112^ Therefore, the M1/M2 ratio has received substantial attention from many scholars. Increasing the proportion of M1-type TAMs induced by IFN-γ can locally restore the M1/M2 ratio and inhibit tumor cell invasion.^126^ In addition, targeting M2-type TAMs with ATRA or coumarin derivatives is a viable alternative.^124^^,^^127^

The immune cells of OS TME are summarized in Table 3.

**Table 3:** Immune cells in the tumor microenvironment of osteosarcoma.

Immune inflammatory cells	T lymphocytes	Peripheral naive T cells induced by signals from the tumor microenvironment	Affect the proliferation and invasion of tumor cells Affect the body's immune function^102^^,^^106^, ^107^, ^108^ Regulate tumor metastasis and spread^105^
	B Lymphocytes	Tumor microenvironment cells and migration from other parts	Affect the proliferation and invasion of tumor cells^114^^,^^105^ Affect the body's immune suppression^113^ Regulate tumor angiogenesis^113^
	Tumor-associated macrophages	Tumor microenvironment cells and migration from other parts	Affect the proliferation and invasion of tumor cells^116^^,^^117^ Regulate tumor metastasis and spread^123^ Disrupt the balance of the body's immune function^118^, ^119^, ^120^ Affect tumor angiogenesis and proliferation^123^

### Neuro interference: cancer-associated nerve cells

The nervous system comprises the peripheral and central nervous systems. Neurological components within TME are part of the nervous system and maintain homeostasis by transmitting information between receptors in the TME and central nervous system.^12^ This process involves both sympathetic and parasympathetic nervous systems. Postganglionic neurons in TME undergo adaptive changes in transcription, translation, and cytoskeletal dynamics owing to the influence of tumors or intrinsic alterations occurring outside the central nervous system.^128^ Studies have suggested reciprocal interactions between sensory nerves and the adrenergic nervous system within TME.^129^ However, given that the cell bodies of postganglionic neurons are located outside the tumor, focusing solely on tumor cells without considering whether the molecular signals of postganglionic neurons inaccurately estimate the impact of nerve cells on the TME is worth exploring.

In clinical settings, patients with bone tumors often present with pain as the primary complaint. The increase in nerve density and tumor progression are mutually reinforcing. However, cancer-induced nerve cell growth is different from nerve-induced cancer cell growth. Cancer cells can promote axonal growth and regeneration. Numerous studies have indicated a close relationship between tumor development and the invasion of sensory nerves in various cancers, including pancreatic cancer, breast cancer, and head and neck squamous cell carcinoma. Moreover, perineural invasion, in which malignant cells invade nerves, serves as a marker of adverse outcomes in cancer and is closely associated with shortened overall survival.

Nerve cells can stimulate tumor progression through feedback loops.^130^ Studies on tumor denervation have reported that nerves within TME can either promote or inhibit tumor development based on the tumor type. Researchers have observed that sensory nerves have been shown to increase the pain threshold and enhance the incidence of tumor metastasis in mouse models. In addition, ablation of nerves can effectively inhibit tumor growth and prolong survival in mice. Adrenergic and sensory nerves predominantly exert pro-tumorigenic effects, whereas the parasympathetic nervous system exerts varying effects based on the cancer type.^131^, ^132^, ^133^ For instance, both sympathetic and parasympathetic nerves predominantly promote tumorigenesis in lung and gastric cancers but exhibit anti-tumor activity in hematological malignancies.^134^, ^135^, ^136^

Recent studies have indicated the existence of a tumor/neuro/immune circuit within TME that mediates communication among cancer cells, immune mechanisms, and the nervous system.^137^ This circuit may help to understand the relationship among stress states, immune function, and cancer cells within TME. Neurological cells in TME can recruit immune cells, activate cancer metastasis, promote lymphocyte apoptosis, and induce macrophage polarization.^138^ Neurological dominance in TME through the inhibition of natural killer cells and cytotoxic T lymphocytes can facilitate tumor progression and metastasis. In addition, up-regulated norepinephrine can guide immune cells to the TME through adrenergic signal transduction.^139^, ^140^, ^141^ Studies on cancer types other than OS have shown that preventing inflammation and neuroinflammatory damage at tumor sites is a promising therapeutic strategy, suggesting that this approach may be effective in inhibiting the proliferation of bone tumors.^142^

### Extracellular vesicles and nucleic acid substances

Traditionally, intercellular communication was thought to occur through direct cell-to-cell contact or the transport of large molecules, such as chemical messengers or hormones. However, recent studies on intercellular communication have revealed a third mechanism underlying the transfer of various substances between cells, including miRNAs, long non-coding RNAs (lncRNAs), and circular RNAs.^143^ This intercellular transfer is mediated by secretory EVs, which originate from different cells and exhibit distinct structures and biochemical characteristics.^144^

### Extracellular vesicles

EVs have been intensively investigated in recent studies. They possess a lipid bilayer spherical structure and express specific protein markers, such as CD63, TSG101, and Alix.^145^ Every cell in the body can secrete EVs based on physiological conditions and control the sorting of their contents. RNA sequencing and proteomic analysis have been used to demonstrate the diversity of contents encapsulated in EVs. EVs can carry both proteins and RNAs and are co-secreted into the ECM, influencing TME and cells within the TME.^146^^,^^147^ Upon entering target cells, EVs release their contents, initiating a cascade reaction in target cells and surrounding organs and consequently influencing tissue transformation. EVs carrying miRNAs can alter the gene expression profiles of receptor cells through gene regulatory mechanisms.^148^ Tumor cell-derived EVs containing miRNAs can modify tumor cell invasion and the transformation of non-tumor cells in TME.^149^^,^^150^

Troyer et al reported that EVs secreted by OS cells differed significantly from those secreted by osteoblasts. OS-derived EVs contained a large number of immunosuppressive proteins, including TGFβ, alpha-fetoprotein, and heat shock proteins.^151^ They not only directly reduced T-cell proliferation but also decreased the expression of CD25 on the surface of CD8^+^ T cells, modulating T-cell immunity. Lin et al showed that miR-501-3p in OS-derived EVs promoted osteoclastogenesis and exacerbated bone loss through both direct and indirect mechanisms.^37^ Li et al found that, compared with osteoblast cell lines, epidermal growth factor receptor (EGFR) phosphorylation-dependent cir-ITCH (circRNA E3 ubiquitin protein ligase) promoted cell migration and invasion, revealing the crucial role of the cir-ITCH/miR-7/EGFR pathway in OS.^152^ Sato et al found that miRNA-218 could target multiple genes, including survivin, in OS, and investigated its impact on the proliferative, migratory, and invasive abilities of OS cells following treatment.^153^

### miRNAs

miRNAs constitute a class of non-coding small RNAs that play diverse roles in cancer.^154^ They mediate the communication between tumor cells and TME components, influencing cancer progression. In the context of OS, the ongoing discovery of new miRNAs, including both anti-cancer and pro-cancer categories, highlights their relevance in cancer development, metastasis, and drug resistance.^155^, ^156^, ^157^, ^158^

Numerous studies have indicated that miRNAs have contradictory effects on the progression of OS cells. Feng et al reported that bone marrow-derived MSCs influenced the proliferative and invasive abilities of OS cells.^159^ miR-877-3p encapsulated in EVs derived from bone marrow-derived MSCs further promoted OS cell migration and vascularization by regulating CREBBP. Shi et al found that EVs, derived from bone marrow-derived MSCs, containing circNRIP1, induced OS through the miR-532-3p/AKT3/PI3K/AKT axis.^160^ Zhang et al showed that the expression of miR-101 was associated with tumor progression in OS and validated the potential anti-cancer effects of miR-101 in *in vivo* models.^101^ Compared with non-metastatic specimens, metastatic OS specimens exhibited reduced expression of miR-101, indicating its inhibitory role in the metastasis of OS. Up-regulated miR-101 directly inhibited the mRNA expression of B-cell lymphoma 6 (BCL6). In addition, viral transduction-mediated systemic administration of EVs derived from adipose tissue-derived MSCs effectively suppressed metastasis *in vivo* with minimal side effects. In addition to influencing metastasis, miRNAs play an essential role in cancer progression. Wang et al found that miR-613 suppressed tumor angiogenesis and proliferation by inhibiting GPR158 (a novel GPR family member associated with the occurrence of glaucoma and cancer) in OS.^161^

### lncRNAs

lncRNAs, like miRNAs, lack protein-coding ability but participate in disease transformation and RNA development.^162^ Numerous studies have indicated that lncRNAs play either oncogenic or tumor-suppressive roles in various cancers, including breast cancer, gastric cancer, colorectal cancer, and hepatocellular carcinoma.^163^^,^^164^ Dysregulation of lncRNAs has been associated with the progression of OS.^165^, ^166^, ^167^

Rothzerg's team conducted a comprehensive investigation into the role of lncRNA in the OS microenvironment. Their findings revealed that RNA sequencing can effectively identify a multitude of up- and down-regulated lncRNAs in OS patient tissues.^168^^,^^169^ For instance, the relative transcript expression of nuclear paraspeckle assembly transcript 1 (NEAT1) was observed to be up-regulated, while neuregulin 1 intronic transcript 1 (NRG1-IT1) expression was found to be down-regulated in OS patient tissues in comparison to normal bone tissues.^169^^,^^170^ Additionally, the researchers were able to ascertain whether the transcript encoded a micropeptide and the location of the transcript within the cell. Zhu et al showed that the lncRNA ODRUL (osteosarcoma doxorubicin-resistance related up-regulated lncRNA) induced the expression of ATP binding cassette subfamily B member 1 (ABCB1) in OS cells, thereby promoting amphotericin resistance and influencing lung metastasis.^165^ ODRUL serves as a competitive endogenous RNA sponge for miR-3182 and promotes the progression of OS by up-regulating MMP2. Wang et al showed that the lncRNA DNACR (differentiation antagonizing non-protein coding RNA) co-regulated ROCK1972 by interfering with miR-1-335p and miR-5 clusters, consequently mediating the growth and transformation of OS cells.^171^ However, Gong et al showed that down-regulation of the lncRNA KIAA0087 promoted the growth, metastasis, and epithelial–mesenchymal transition of OS cells by targeting the suppressor of cytokine signaling 1 (SOCS1)/JAK2/STAT3 pathway mediated by miR-411-3p.

These findings suggest that lncRNAs do not exhibit pro-cancer effects uniformly.^166^ In recent years, the biological role of lncRNAs in cancer has received widespread attention, and existing evidence suggests that dysregulation of lncRNAs leads to the malignant progression of OS.

EVs and nucleic acid substances of OS TME are summarized in Table 4.

**Table 4:** Extracellular vesicles and nucleic acid substances in the tumor microenvironment of osteosarcoma.

Extracellular vesicles		Tumor cells and microenvironment cells	Carry contents facilitate the material exchange to promote or suppress cancer^146^^,^^148^, ^149^, ^150^, ^151^
Nucleic acid substances	miRNA	Tumor cells and microenvironment cells	Affect the proliferation and invasion of tumor cells^159^ Regulate tumor metastasis and spread^161^ Inhibit the body's immune function Inhibit tumor angiogenesis and proliferation^161^
	lncRNA	Tumor cells and microenvironment cells	Participate in disease progression and RNA development^162^ Affect the proliferation and invasion of tumor cells^171^ Regulate tumor metastasis and spread Target tumor epithelial–mesenchymal transition progression^166^

## Summary and prospects

Since the introduction of TME, many researchers have identified a close relationship between tumor progression and TME. The diversity of TME components results in distinct physiological features of TME, such as hypoxia, acidic niches, neural innervation, and microenvironmental inflammation. The numerous cellular and non-cellular components present within TME are closely associated with the malignant progression of tumors, influencing immune tolerance, drug resistance, and metastasis.^172^^,^^173^ M2-type TAMs contribute to the growth of OS by promoting angiogenesis, facilitating immune tolerance in T lymphocytes, and participating in the transport of EVs.^124^^,^^174^ In recent years, significant progress has been made in improving prognostic outcomes using therapies targeting the TME components of OS. The study of TME and its relationship with the development of OS has important clinical implications for the diagnosis, prognostic assessment, and optimization of treatment strategies for OS. This review summarized the roles of TME components in the progression of OS and discussed the existing therapeutic approaches. TME-specific treatment strategies can selectively target TME components, such as bone-related cells, immune cells, and exosomes. Novel biomaterials targeting TME components can be developed to inhibit the progression of OS. The composition of TME varies across different locations in the body and among patients with OS. Developing TME-targeting therapies is challenging owing to the complexity and heterogeneity of samples and close interactions among immune cells. To identify and target specific components, a more comprehensive system-level approach should be used to analyze and integrate all TME components instead of focusing on individual target cell types. However, the long-term use of TME-specific targeted therapies and immunotherapies may lead to resistance, as in the case of chemotherapy. A comprehensive grasp of the mechanisms of action of TME components can inform the development of more targeted and immunotherapeutic strategies, particularly in the context of drug resistance and tumor metastasis. The study of TME-specific therapeutic strategies not only provides new ideas to inhibit the malignant progression of OS but also establishes the basis for the development of TME-based biomarkers and multimodal combination therapies, which can help to improve the overall survival and quality of life of OS patients. Furthermore, elucidating the heterogeneity and dynamic alterations of the TME will assist clinicians in formulating more efficacious treatment regimens tailored to the individual characteristics of each patient, thereby enhancing prognostic outcomes. Consequently, utilizing multiple approaches to alleviate immune suppression and exploring the combination of various treatment modalities may yield meaningful clinical outcomes. Despite remarkable progress in research, the characterization of OS cells and the TME of OS remains incomplete. Therefore, future studies should aim to identify valuable immune markers within TME to guide the development of more effective targeted therapies and immunotherapies for OS.

## CRediT authorship contribution statement

**Yihan Yu:** Writing – original draft. **Kanglu Li:** Conceptualization. **Yizhong Peng:** Writing – review & editing. **Zhicai Zhang:** Supervision. **Feifei Pu:** Conceptualization. **Zengwu Shao:** Investigation. **Wei Wu:** Writing – review & editing.

## Funding

This work was supported by the 10.13039/501100001809National Natural Science Foundation of China (No. 82274559, 81904231).

## Conflict of interests

The authors declared no potential conflict of interests.
